# Heat Extremes Driven by Amplification of Phase‐Locked Circumglobal Waves Forced by Topography in an Idealized Atmospheric Model

**DOI:** 10.1029/2021GL096337

**Published:** 2022-11-09

**Authors:** B. Jiménez‐Esteve, K. Kornhuber, D. I. V. Domeisen

**Affiliations:** ^1^ ETH Zürich Institute for Atmospheric and Climate Science Zürich Switzerland; ^2^ Earth Institute Columbia University New York NY USA; ^3^ Institute of Earth Surface Dynamics University of Lausanne Lausanne Switzerland

**Keywords:** topography, circumglobal teleconnections, Rossby waves, heatwaves, idealized modeling, phase locking

## Abstract

Heatwaves are persistent temperature extremes associated with devastating impacts on human societies and ecosystems. In the midlatitudes, amplified quasi‐stationary Rossby waves have been identified as a key mechanism for heatwave occurrence. Amplified waves with preferred longitudinal locations lead to concurrent extremes in specific locations. It is therefore important to identify the essential components in the climate system that contribute to phase‐locking of wave patterns. Here, we investigate the role of dry atmospheric dynamics and topography in causing concurrent heatwaves by using an idealized general circulation model. Topography is included in the model experiments as a Gaussian mountain. Our results show that amplified Rossby waves exhibit clear phase‐locking behavior and a decrease in the zonal phase speed when a large‐scale localized topographic forcing is imposed, leading to concurrent heat extremes at preferred locations.

## Key Points

- The inclusion of topography in an idealized model setup yields global‐scale slow‐moving Rossby waves with preferred longitudinal locations
- When these slow‐moving Rossby waves amplify they lead to an increased frequency of concurrent heatwaves in preferred longitudinal locations
- In the model, zonal wavenumbers 5 and 6 exhibit the strongest anomalies, which strongly impact temperature and heatwave frequency

## 1 Introduction

Heatwaves remain one of the deadliest geophysical events (Hoegh‐Guldberg et al., 2018; Koppe et al., 2004; Yaghmaei, 2020) and are projected to increase in magnitude and frequency with climate change (Lopez et al., 2018; Perkins et al., 2012; Perkins‐Kirkpatrick & Lewis, 2020; Watanabe et al., 2013). A thorough understanding of the physical driving mechanisms is necessary to improve their prediction and to identify regions at risk under future emission scenarios. High impact extreme weather events in the Northern Hemisphere midlatitudes can be associated with slow moving, that is, quasi‐stationary, and high‐amplitude Rossby waves, which can remain in place for several days to weeks (Petoukhov et al., 2013). Most notable examples include the European heatwaves of 2003 (Kornhuber, Petoukhov, Petri, et al., 2017), 2015 (Duchez et al., 2016) and 2018 (Drouard et al., 2019; Kornhuber et al., 2019; Vogel et al., 2019; Wehrli et al., 2020), the Russian heatwave of 2010 (Di Capua et al., 2021; Lau & Kim, 2012), and the recent North American heatwave of 2021 (Philip et al., 2021). Such extreme weather events often do not occur in isolation, but are a regional imprint of a hemispheric pattern with extreme heat and rainfall extremes occurring almost simultaneously in different longitudinal locations (Coumou et al., 2014; Kornhuber et al., 2020). For instance, the circumglobal wave pattern that contributed to the 2018 European heatwave also promoted extreme rainfall and flooding in Greece, a heatwave in the Caspian Sea region and extreme rainfall in Japan (Kornhuber et al., 2019), as well as a concurrent heatwave in California (Domeisen et al., 2022). The amplified wave pattern that fueled the extreme heat over northwestern North America in late June 2021 occurred almost simultaneously with an extreme heatwave in Scandinavia and Siberia.

Topography and thermal contrast, for example, across land—ocean boundaries, act on the atmospheric circulation and favor particular wave patterns of regional and hemispheric extent that exhibit a strong seasonal cycle (Chang, 2009; Charney & Eliassen, 1949; Held et al., 2002). For example, the Rocky Mountains force a stationary ridge over the North American continent (e.g., Brayshaw et al., 2009; Saulière et al., 2012), which might have strengthened in recent winters (Singh et al., 2016). Similarly, the subtropical jet over Eurasia is influenced by the Tibetan plateau and Mongolian mountains (White et al., 2017) and exhibits a recurrent flow pattern called the *Silk road pattern*, which is associated with co‐variability ranging from Europe to east Asia (Boers et al., 2019; Ding & Wang, 2005; Kosaka et al., 2009; Lu et al., 2002). Given sufficiently strong forcing and suitable background conditions (also known as “waveguidability,” see Manola et al. (2013); Wirth (2020); Wirth and Polster (2021); White et al. (2021)), Rossby waves can form circumglobal teleconnections (Branstator, 2002; Teng & Branstator, 2019). As the location and strength of the background flow changes seasonally, different circumglobal teleconnection patterns have been identified in summer and winter (Branstator, 2002; Ding & Wang, 2005; Kornhuber et al., 2020). In summer, recurrent wave 5 and 7 patterns have been identified that cause concurrent heatwaves in specific regions in the Northern Hemisphere midlatitudes (Kornhuber et al., 2020). These wave patterns have been detected during some of the most extreme heat events of recent years: The European heatwaves of 2003 and 2018 have been diagnosed as a wave 7 pattern (Kornhuber et al., 2019), while the 2010 heatwave was associated with a wave 5–6 pattern (Figure 2 in Petoukhov et al. (2013) and Di Capua et al. (2021)).

Several mechanisms have been suggested to cause such amplified wave patterns. Quasi‐resonant amplification of planetary waves (Petoukhov et al., 2013) describes the process during which a waveguide forms under specific zonal mean flow conditions (Manola et al., 2013), which allows waves to amplify given sufficient forcing. Studies investigating the amplification of circumglobal wave patterns and phase‐locking behavior, that is, the fact that under stationary forcing waves of certain wavenumbers exhibit preferred longitudinal locations, so far are based on reanalysis data (e.g., Kornhuber, Petoukhov, Karoly, et al., 2017; Kornhuber, Petoukhov, Petri, et al., 2017; Petoukhov et al., 2018) or more complex atmospheric general circulation models (Huntingford et al., 2019), for which it can be challenging to isolate specific dynamical mechanisms. Thus the analysis of wave amplification and phase‐locking of amplified waves under more idealized conditions is still needed for process understanding (White et al., 2022). Here, we investigate the role of topographic forcing in idealized model experiments, where only basic atmospheric dynamics are represented together with topography. This model setup allows us to focus on how localized topography affects the propagation of midlatitude Rossby waves and influences the preferred location where troughs and ridges associated with circumglobal patterns amplify and lead to an increased likelihood of temperature extremes. This approach further clarifies the interaction between orographically forced Rossby waves and transient Rossby waves originating from baroclinic instability.

## 2 Data and Methods

### 2.1 Model Setup

We use an idealized configuration of the ICOsahedral Nonhydrostatic (ICON) atmospheric model (Zängl et al., 2015) on a *R*_2_ *B*_4_ triangular grid, with an equivalent ∼158 km grid spacing, and 41 vertical levels. We turn off all the physical parameterizations and substitute them by a simple zonal mean Newtonian temperature relaxation parameterization as in Held and Suarez (1994). A more detailed description of the model setup can be found in Jiménez‐Esteve and Domeisen (2022).

Idealized topography is introduced in the form of a 2‐dimensional Gaussian mountain with a maximum height *H* at a latitude and longitude of (*ϕ*_0_, *λ*_0_ = 90°E). The half‐widths of the mountain are fixed to *σ*_*x*_ = *σ*_*y*_ = 1,500 km. The mathematical formulation for the topography follows Cook and Held (1992), except that in our case the half‐widths are given in km (instead of °), which preserves the volume of the mountain when changing its latitude. We use three topography configurations: (*H*, *ϕ*_0_) = (4 km, 45°N), (*H*, *ϕ*_0_) = (8 km, 45°N), and (*H*, *ϕ*_0_) = (8 km, 25°N). Each simulation is run for a total of 30 years (360 days a year) using the same equilibrium temperature and relaxation timescale as in Held and Suarez (1994).

The zonal wind climatology at 300 hPa (u300) of the model simulations and ERA‐Interim reanalysis (Dee et al., 2011) are shown in Figure S1 in Supporting Information S1. It is important to note that our model does not simulate a seasonal cycle, and background conditions are close to the observed annual mean conditions.

### 2.2 Zonal Wavenumber Decomposition, Phase‐Locking, and Hayashi Spectra

We use the meridional wind at 300 hPa (v300) to identify Rossby waves in the midlatitude troposphere. In an idealized framework a Rossby wave can be mathematically written as a perfect zonal sinusoidal circumglobal wave:

(1) v300(λ,t)=A(t)cos(kλ+Φ(t))

where *λ* is the longitude in radians, *t* the time, *A*(*t*) > 0 is the amplitude of the wave, *k* = 1, 2, 3, … the non‐dimensional zonal wavenumber, Φ(*t*) is the phase of the wave in radians at time *t*.

At every given time *t* the v300 field can be decomposed into a range of superimposed cosine waves of different wavenumbers *k*, with amplitudes *A*_*k*_ and phases Φ_*k*_. We first calculate the area‐weighted latitudinal mean between 60° and 30°N and then apply a 7‐day running mean to the v300 field in order to filter out medium‐ and high‐frequency transient waves. Then, a Fast Fourier Transformation in the longitude dimension is applied to obtain the amplitude (*A*_*k*_) and the phase (Φ_*k*_) for each wavenumber *k*. It is important to highlight that for our wave amplitude and phase calculation we do not subtract the model climatology. Temporal wave anomalies cannot have a phase preference as by definition the time average must be zero at all points. The climatology and percentiles of the obtained amplitudes for each simulation and reanalysis can be found in Figure S2 in Supporting Information S1 for reference. We focus on wavenumbers *k* = 4–8, since synoptic‐scale waves are commonly involved in circumglobal wave amplification events, for example, *k* = 4 (Petoukhov et al., 2018), *k* = 5 (Harnik et al., 2016; Teng et al., 2013), or *k* = 6–8 (Mann et al., 2017; Petoukhov et al., 2013, 2016), and exhibit phase locking behavior in reanalysis (Kornhuber, Petoukhov, Petri, et al., 2017).

To study the high‐amplitude episodes that are characterized by single (phase‐locked) wavenumbers, we calculate composites by averaging the unfiltered v300 anomalies, temperature at 1,000 hPa (T1000) anomalies (with respect to the same model run) and heatwave frequency anomalies (see Section 2.3 for definition) over time steps when *A*_*k*_ > 1.5 standard deviations (SDs) for each specific wavenumber *k*. To analyze the temporal evolution of high‐amplitude events, we define the onset of a wave amplification event as the first day of at least three consecutive days of *A*_*k*_ > 1.5*SD* for each wavenumber *k*.

To quantify phase‐locking we define the index *δ*_*m*_ as the narrowest window that contains half of the probability density of the phase distribution. Lower values of *δ*_*m*_ correspond to stronger phase‐locking. To estimate the phase speed *c* of the waves, we compute the Hayashi spectrum (Hayashi, 1979) using the methodology by Randel and Held (1991). For an application of this diagnostic see Domeisen et al. (2018) and Riboldi et al. (2021). A more detailed description of these two methodologies can be found in Supporting Information S1. In this study we use the term “quasi‐stationary” waves to refer to wave anomalies that are slow moving, that is, their phase‐speed is close to zero, while the term “stationary” waves is used to refer to the climatological waves.

### 2.3 Heatwave Definition

Heatwaves are identified as periods of three or more consecutive extreme heat days, which are defined as days when the temperature at the 1,000 hPa pressure level (T1000) is above the 95th percentile. Other pressure levels close to the surface yield comparable results. The 95th percentile is computed independently for each grid point and for each model experiment using unfiltered data. We do not consider a minimum temporal separation between events. The heatwave frequency is defined as the fraction of days that belong to heatwaves.

## 3 Results

**Figure 1:**
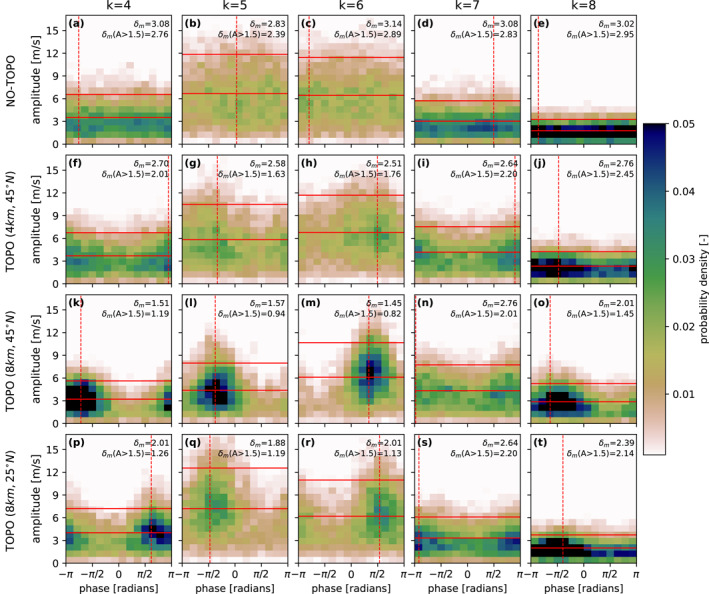
Two‐dimensional histograms of wave amplitude versus phase of the meridional wind at 300 hPa (v300) for the model experiment (a–e) without topography (no‐topo), (f–j) with 4 km topography centered at (45°N, 90°E), (k–o) with 8 km topography centered at (45°N, 90°E), and (p–t) 8 km topography centered at (25°N, 90°E). Each column shows a different zonal wavenumber (*k* = 4–8). The mean and the 1.5 SD amplitude threshold are indicated as horizontal red lines. The vertical dashed line indicates the phase of maximum probability for all amplitudes. The phase‐locking index (*δ*_*m*_ in radians) is displayed in the upper‐right corner of each panel, also for the high‐amplitude cases (a lower *δ*_*m*_ indicates a stronger phase‐locking for a particular zonal wavenumber). A total of 10,794 values (30 years of 360 days each, where the first and last 3 days are disregarded due to the 7‐day running mean window) are used to produce each of the histograms. No smoothing is applied.

To quantify which wavenumbers exhibit stronger phase‐locking when topography is included in the model, we look at the 2‐dimensional distribution of the amplitude versus phase for each zonal wavenumber (Figure 1). See Section 2.2 for details on the calculation. For the simulation without topography (no‐topo) (Figures 1a–1e) zonal wavenumbers *k* = 4–8 indicate no preference for phase‐locking. In other words, when waves amplify, that is, when their wave amplitude increases to above average values, their maxima can occur with equal probability in any longitudinal location. In contrast, in the experiments with an 8 km mountain located in midlatitudes (45°N, Figures 1k–1o) or in the subtropics (25°N, Figures 1p–1t) waves are more likely to amplify for a specific phase, that is, longitudinal location, meaning that they are phase‐locked. The same effect but of weaker intensity can be observed for the smaller 4‐km mountain (Figures 1f–1j). Phase‐locking does not occur with the same amplitude for the different synoptic wavenumbers, and it is particularly strong for wavenumbers 4, 5 and 6 (see lower values of *δ*_*m*_ in Figure 1 and Figure S3 in Supporting Information S1). Large amplitude events (*A*_*k*_ > 1.5*SD*) tend to exhibit higher values of phase‐locking for extreme amplitude events compared to climatology, consistent with results found by Kornhuber et al. (2020) using reanalysis data. For the remainder of this study we will predominantly focus on wavenumbers 5 and 6, as those wavenumbers exhibit higher amplitudes than wavenumber 4 in our model configuration, while other wavenumbers are discussed in less detail. Larger synoptic wavenumbers (*k* > 6) usually exhibit weaker phase locking and maximum amplitudes and thus their impact on extreme heat events is expected to be less important. Note that the higher amplitudes for waves 5 and 6 can also be observed for the no‐topo experiment (Figures 1b and 1c, Figure S2 in Supporting Information S1), and is probably a consequence of baroclinic instability in our model configuration (Grotjahn, 2015). Note that there is no asymmetric forcing that can force waves in the no‐topo run and thus the largest amplitudes found for wavenumbers 5 and 6 are independent of topographic forcing.

**Figure 2:**
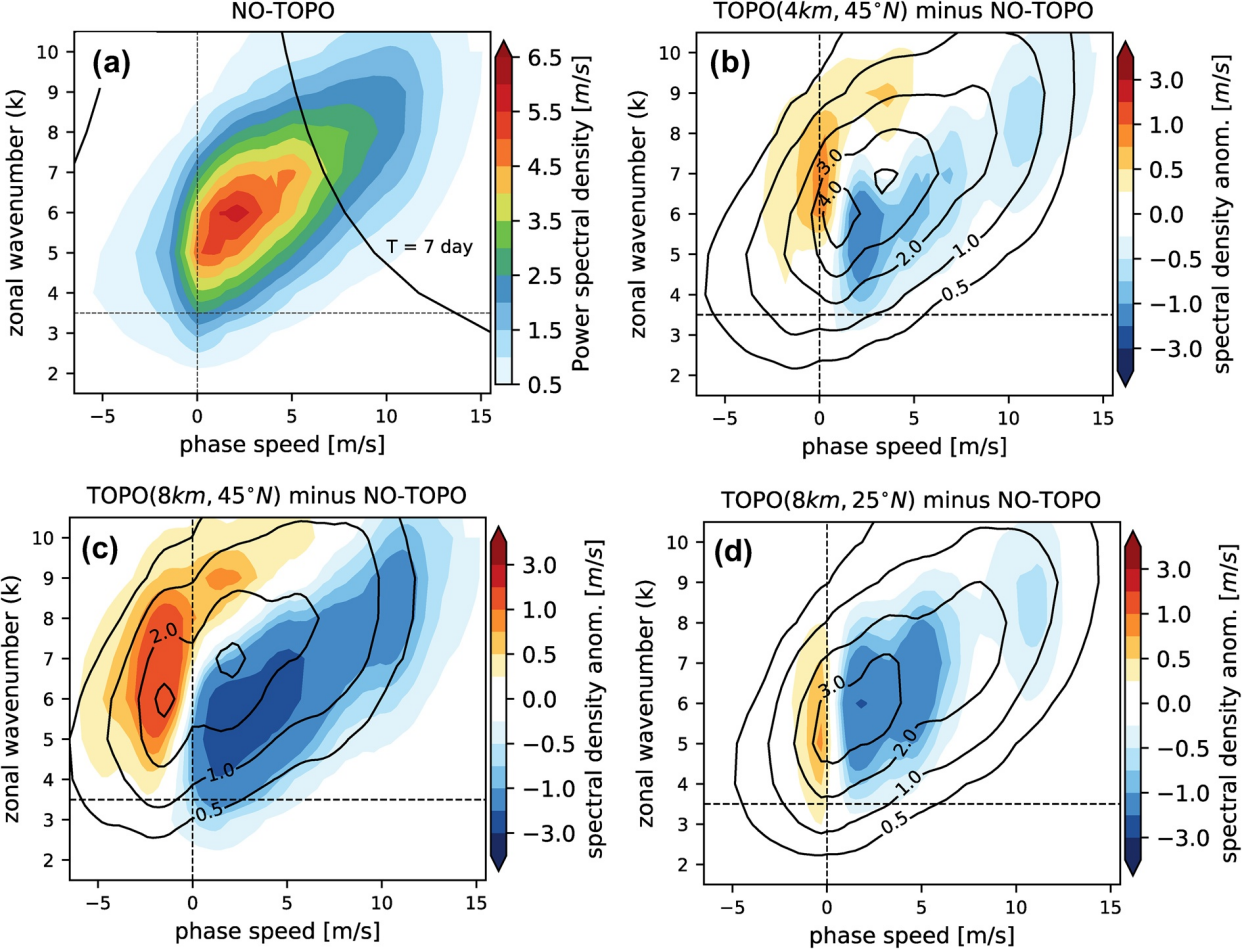
Hayashi spectra of v300 anomalies (m/s) as a function of phase speed (m/s) relative to the ground and zonal wavenumber *k* for (a) the no‐topography simulation (shading) and (b–d) three different topographic forcings (black contours). The difference between the spectral densities of each topography experiment and the no‐topo experiment is shown as color shading in panels (b–d). The black line in panel (a) indicates a constant period of 7 days for reference.

As a next step, we examine how topography impacts the phase speed of waves. The Hayashi spectrum (see Section 2.2 and Supporting Information S1) allows us to quantify the distribution of the power spectrum for the midlatitude flow in terms of zonal wavenumber and phase speed (Figure 2). Note that we do not apply any temporal filter to the data in order to represent the full spectrum of waves. For the no‐topo run the spectral density is maximized for synoptic wavenumbers *k* = 4–7 with a peak around *k* = 5, 6 (Figure 2a), consistent with the maximum amplitudes observed in Figure 1 and Figure S2 in Supporting Information S1. A similar spectrum is found for reanalysis during equinoxes (Riboldi et al., 2021), which highlights the ability of our model to simulate realistic atmospheric conditions. Figure 2a also shows how larger wavenumbers tend to be associated with faster eastward propagation, which is a consequence of the Rossby wave dispersion relation (*c* = *U* − *β*/(*k* ^2^ + *l* ^2^)). Thus, small wavenumbers (*k* < 7) can more often become quasi‐stationary (slow‐moving) if the mean flow in the midlatitude troposphere slows down sufficiently.

We now investigate the effect of localized topographic forcing on the Hayashi spectra (Figures 2b–2d). Already a moderate forcing (4 km, 45°N) has an effect on the spectra, with an increasing power of quasi‐stationary waves, especially for wavenumbers 6–8, and decreasing eastward propagating synoptic waves with respect to the no‐topo run (Figure 2b). This effect is stronger for a doubled mountain height located at the same latitude (8 km, 45°N), with a clear decrease in the eastward phase speed spectral density (Figure 2c). For a subtropical topography forcing (8 km, 25°N) there is also a decrease in eastward propagating synoptic waves, while slow‐moving westward propagating waves are more common than in the no‐topography simulation (Figure 2d).

**Figure 3:**
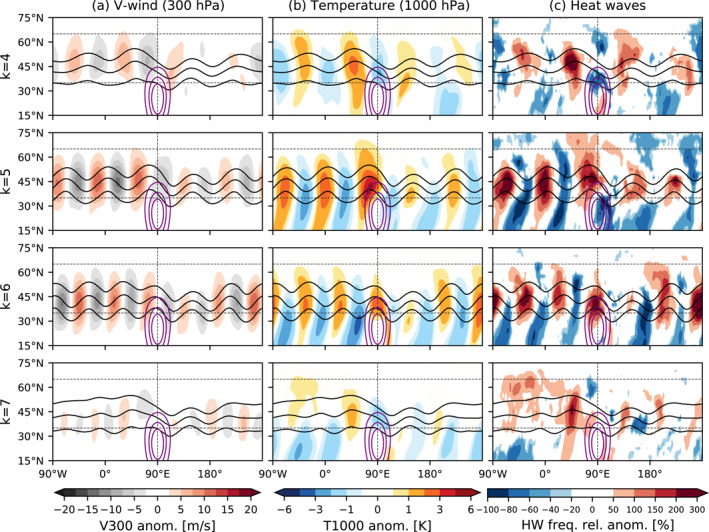
Mean composites of (a) anomalies of meridional wind at 300 hPa (v300), (b) anomalies of air temperature at 1,000 hPa (T1000), and (c) heatwave frequency relative anomaly (note the nonlinear colorbar [−100 to 0 in 20% intervals; 0 to 200 in 50% intervals]) for high‐amplitude (*A* > 1.5 SD) days of zonal wavenumbers *k* = 4–7 (top to bottom) identified in the 8 km and 25°N topography experiment. In each panel, the geopotential height at 300 hPa is displayed as black contours (8,800, 9,000, and 9,200 m) and topographic elevation in purple contours (1, 3, and 5 km). Note that geopotential height contours at smaller distances correspond to a stronger geostrophic wind. The heatwave frequency relative anomaly shown in panel (c) is the percentage change in heatwave frequency relative to the climatological frequency of heatwaves in each model simulation. Only statistically significant anomalies above the 95% confidence level are shown according to a two‐tailed *t*‐test.

To analyze the impact on near‐surface temperature and persistent heat extremes associated with amplification of phase‐locked waves we show composites of the unfiltered v300, T1000 and the heatwave frequency relative anomaly for all days when a high amplitude of a specific wavenumber is identified in the (8 km, 25°N) topography simulation (Figure 3). Note that the waves shown in Figure 3 are anomalies with respect to the model's zonally varying climatology, that is, they are in addition to the stationary waves excited by topography. Qualitatively similar results are obtained for the midlatitude forcings (Figures S4 and S5 in Supporting Information S1), although we find that the response for the subtropical mountain exhibits a more pronounced wave pattern of near‐hemispheric extent. Figure 3 confirms the circumglobal and phase‐locked character of the high‐amplitude events identified in our simulations, which can be seen as a series of wind anomalies of alternating sign extending to the full longitude circle in midlatitudes (Figure 3a). Note that the circumglobal characteristic for wavenumbers 5 and 6 is not just a result of the multi‐event average (see Figure S8 in Supporting Information S1). The same analysis with the no‐topo run (not shown) leads to negligible anomalies, as although the maximum amplitudes can be similar they do not occur with a preferred phase (Figures 1a–1e). The composited anomalies have approximately twice the magnitude for *k* = 5 and 6 as compared to *k* = 4 and 7, consistent with Figures 1 and 2. Associated with the upper level troughs and ridges (black contours in Figure 3) T1000 anomalies show a circumglobal pattern, which is approximately 90° out of phase with the v300 anomaly field. Interestingly, T1000 and v300 anomalies are stronger upstream than downstream of the topography (less clear for *k* = 7). This weaker response downstream is likely a consequence of a stronger zonal jet speed (Figure S1 in Supporting Information S1), which is related to the stronger meridional temperature gradients downstream of large‐scale topography (e.g., Lutsko et al., 2019).

The effect of circumglobal wave amplification on heatwave occurrence is also shown in Figure 3c. Consistent with the temperature anomalies near the surface, heatwave frequency increases in the regions with atmospheric ridges, associated with a relative increase of up to 300% mainly for wavenumbers 5 and 6. This means a heatwave is four times more likely to occur during a wave amplification event than for climatological conditions in this specific longitudinal location. In locations where troughs amplify we observe a clear decrease in the probability of having a heatwave of up to a 100%.

**Figure 4:**
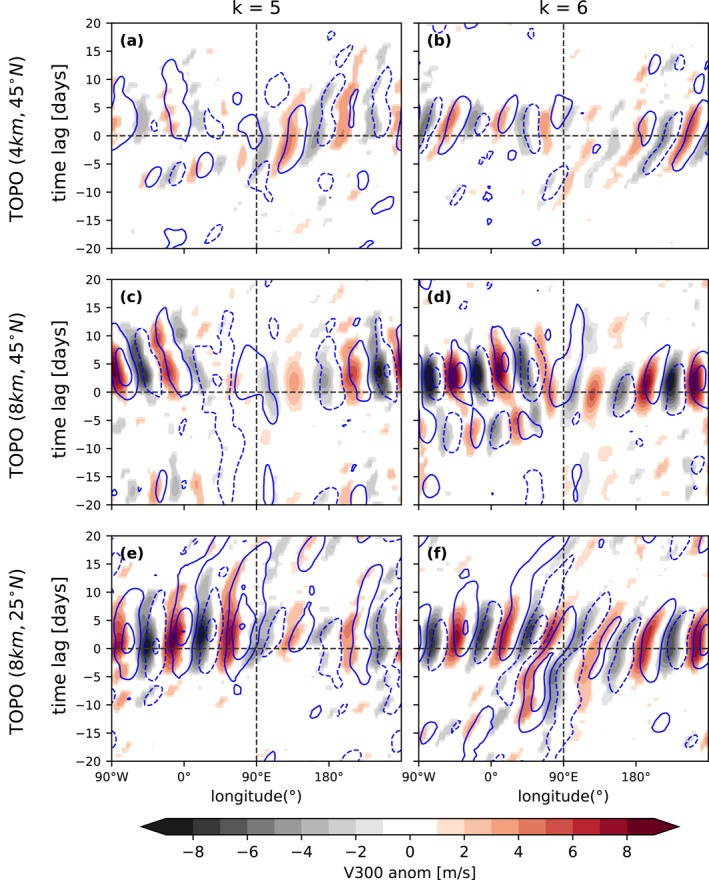
Hovmöller diagram (time vs. longitude) of daily mean v300 anomalies (shading) and T1000 anomalies (blue contours: from −2.5 to 2.5 in 1 K intervals, negative dashed) averaged over 30°–60°N and composited with respect to the onset of high‐amplitude (a, c, d) *k* = 5 and (b, d, f) *k* = 6 events. (a and b) 4 km mountain at 45°N, (c and d) 8 km mountain at 45°N, and (e and f) 8 km mountain at 25°N. Only statistically significant v300 anomalies above the 95% confidence level are shown according to a two‐tailed *t*‐test.

To gain a deeper insight into the dynamical evolution of wave amplification events, we compute the multi‐event average of v300 and T1000 with respect to the onset date (see Section 2.2). Figure 4 displays the temporal evolution of the v300 and T1000 anomalies averaged over 30°–60°N for amplification events of *k* = 5 and 6 in the three model simulations. We display the results for wavenumbers 5 and 6 as those show the strongest amplitudes and more phase‐locking in our model simulations (Figure 1). The results for *k* = 4,7 are shown in Figure S6 in Supporting Information S1. The amplitudes of wavenumber 5 and 6 strongly dominate the signal during amplification events, while this is not the case for the amplification of other zonal wavenumbers, as their amplitudes are in general much weaker than the amplitudes of the dominant wavenumbers (*k* = 5, 6) (see Figure S8 in Supporting Information S1).

The Hovmöller diagram (Figure 4) shows that wave amplification events tend to last on average 6–7 days for both *k* = 5 and 6 (see also Figure S9 in Supporting Information S1). The quasi‐stationary wave anomalies (with respect to the zonally varying model climatology) show a similar phasing as the climatological stationary waves generated by topography (Figure S7 in Supporting Information S1). It is interesting to note the westward phase speed for the disturbances located upstream of the mountain peak (mainly for *k* = 5 in the TOPO [8 km, 45°N] experiment). This westward phase speed is likely due to slower zonal winds upstream than downstream of the mountain (Figure S1 in Supporting Information S1) and due to the Rossby wave dispersion relation, which states that wavenumber 5 propagates faster westward with respect to the mean flow than wavenumber 6. Note that the stronger v300 anomalies in the averaged composite for TOPO (8 km, 45°) compared to TOPO (4 km, 45°) result from a more intense phase‐locking for the stronger forcing (Figure 1). When the topography is located south of the jet axis (Figures 4e and 4f), v300 anomalies associated with *k* = 5, 6 wave amplification tend to be more circumglobal (a similar magnitude for all longitudes) than for the midlatitude mountain. The phase speed (tilt of the v300 anomalies) during these events tends to be close to zero, especially for *k* = 5 and upstream of the mountain, where zonal winds tend to be weaker (Figure S1 in Supporting Information S1).

Another important question is whether there are clear precursors to wave amplification events. Rossby wave packets (Wirth et al., 2018) of limited longitudinal extent develop up to 15–20 days before the onset of some wave amplification events, with energy propagating eastwards (positive group velocity). These precursors are clearer in some of the composites, see for example, Figures 4a and 4d (between 90°W and 90°E and 0–10 days before the onset). For *k* = 6 and a subtropical mountain (Figure 4f) v300 anomalies emerge even earlier and amplify 15 days before the onset near the location of the mountain before the initiation of the circumglobal response. Future work should investigate the relationship between the Rossby wave packets and the circumglobal wave amplification.

## 4 Discussion and Conclusions

In this study, we show evidence that localized topography leads to phase‐locked amplification of quasi‐stationary synoptic‐scale waves. We base our analysis on idealized simulations using only thermal forcing and Rayleigh surface drag as in (Held & Suarez, 1994), with no other physical parameterizations, which allows us to demonstrate that very few “ingredients” are needed to produce phase‐locked and amplified waves and associated concurrent heatwaves. Observed phase‐locking events have been shown to be associated with simultaneous extreme events over major crop regions across the globe (e.g., Kornhuber et al., 2020). Our results confirm that a similar connection between heatwaves and phase‐locked amplified waves exists in a simple model configuration, originating solely from topographic forcing and dry atmospheric dynamics.

These experiments highlight that changes in the relative position of the mean flow with respect to the topographic disturbance can lead to different responses in terms of the amplitude of circumglobal phase‐locked waves, which impacts the frequency and location of heatwave hotspots (cf., Figure 3 and Figure S3 in Supporting Information S1). Observations show a weak poleward shift of the midlatitude jet (Osman et al., 2021) that is projected to continue under high emission warming scenarios (Harvey et al., 2020). This shift results in a different location of the jet with respect to the topographic forcing, although this relative shift between the jet and the topography is considerably smaller on average than in our idealized simulations. Nevertheless, our results suggest that a jet shift might lead to a change in future heatwave hotspots. While past discussions on atmospheric circulation changes and impacts on summer weather extremes have put a primary focus on changes in the jet speed (Coumou et al., 2015; Kornhuber & Tamarin‐Brodsky, 2021) and its ability to support resonant waves (Mann et al., 2018), our results suggest that changes in jet latitude relative to important mountains should be given similar attention. Apart from the location, the shape and height of the topographic forcing can impact the stationary wave response (Brayshaw et al., 2009; Valdes & Hoskins, 1991). In this study, we keep the shape of the mountain fixed to a half‐width of 1,500 km, which is comparable to the scale of the Tibetan plateau or the Rocky mountains (see Figure S1 in Supporting Information S1). Moreover, nonlinear interactions with other mountain ranges (Narinesingh et al., 2020) and diabatic forcing originating from land‐sea contrast and tropical convection (Garfinkel et al., 2020; Kaspi & Schneider, 2011) modulate the total stationary wave response. Using a simplified version of topography allows us to clearly identify the main role of topographic forcing.

While this analysis focuses on the relationship between topographic forcing and phase‐locking, the forcing leading to circumglobal teleconnections can be more diverse under real‐world conditions (Branstator, 2002; Teng & Branstator, 2019). Apart from the topographic forcing investigated here, thermal forcing, for example, from land‐sea thermal contrast, can be responsible for preferred positions of Rossby waves. In addition, phase‐locked wave trains can arise from SST anomalies (Beverley et al., 2021) or dry soil conditions over large continental areas (Teng et al., 2019).

Our work relies on a constant thermal forcing throughout the experiments, and thus there is no long‐term trend or seasonal cycle. However, it has been suggested that waveguide characteristics might change in a warmer climate potentially favoring wave amplification (Mann et al., 2017, 2018). Although a recent review by Teng and Branstator (2019) concludes that changes in the diabatic heating are a more probable candidate for more high‐amplitude circumglobal planetary wave events in the future, reported changes in the zonal mean circulation for example, from an overall slow‐down (Coumou et al., 2015) or strengthening of the jet in certain latitudes (Xu et al., 2020) will likely affect the waveguiding ability of the zonal jet.

Impacts of heatwaves are strongly amplified with their increased persistence. While topography plays an essential role in determining the longitudinal structure of circumglobal wave patterns, its presence does not lead to a higher persistence of the high‐amplitude events and might even lower their persistence in some cases (Figure S9 in Supporting Information S1). Hence, other components of the climate system not included in our idealized experiments may be responsible for an increased persistence of heatwaves associated with amplified wave patterns. Important mechanisms include land‐surface feedbacks (Miralles et al., 2019; Schumacher et al., 2019; Teng et al., 2019) and diabatic heating maintaining upper level blocking anticyclones (Pfahl et al., 2015; Zschenderlein et al., 2020). Further work is required to improve our understanding of the relative importance of the different processes that drive amplification, phase‐locking and maintenance of amplified waves. Models struggle with an accurate simulation of circumglobal wave patterns (e.g., Luo et al., 2021) and the magnitude of associated weather extremes. Understanding how the stationary components of the climate system (such as topography) interact with those components that might be affected by climatic change (such as land‐atmosphere interactions and the circulation itself) is important for reducing model biases in order to improve our ability to predict and project changes in concurrent extreme events such as heatwaves in current and future climates.

## Supporting information

Supporting Information S1

## Data Availability

The ERA‐Interim reanalysis (Dee et al., 2011) has been obtained from the ECMWF server (https://apps.ecmwf.int/datasets/data/interim-full-daily/levtype=pl/, last access: 27 September 2021). All analyses and visualizations were carried out using the Python packages NumPy (https://numpy.org/, last access: 21 June 2022), Matplotlib (https://matplotlib.org/, last access: 21 June 2022), xarray (https://docs.xarray.dev/en/stable/, last access: 21 June 2022), and SciPy (https://scipy.org/, last access: 21 June 2022). The code to reproduce the analysis and the main figures of this paper can be found in GitHub (https://github.com/bernatj/paper_GRL_phase_locked_circumglobal_heat_extremes.git). The daily mean model output is available in zenodo (https://doi.org/10.5281/zenodo.7270361).
