# Supplementary material for: Heat Extremes Driven by Amplification of Phase‐Locked Circumglobal Waves Forced by Topography in an Idealized Atmospheric Model
Source: Geophys Res Lett. 2022 Nov 9;49(21):e2021GL096337. doi: 10.1029/2021GL096337 (PMC9787382; doi:10.1029/2021GL096337)
Supplement: Supplementary file 1 — Supporting Information S1 [file GRL-49-e2021GL096337-s001.pdf]

Supporting Information for "Heat extremes driven by amplification of phase-locked circumglobal waves forced by topography in an idealized atmospheric model"

B. Jiménez-Esteve[1], K. Kornhuber[2], D. I.V. Domeisen[1,3]

[1]ETH Zurich, Institute for Atmospheric and Climate Science, Zurich, Switzerland

[2]Earth Institute, Columbia University, New York City, USA

[3]University of Lausanne, Institute of Earth Surface Dynamics, Lausanne, Switzerland

Contents of this file

1. Figures S1 to S9

Introduction

The supporting information provides more details about the model climatology and the methods employed in this study. It also contains supplementary figures to support some of the conclusions in the main manuscript.

The model zonal wind climatology at 300h Pa (u300) for the 4 model experiments presented in the study this shown alongside with the ERA-Interim reanalysis in Figure S1. The variability in the wave amplitude is shown as box plots for different zonal wavenum-

bers for the model runs and reanalysis is shown in Figure S2. We also include the same composite analysis of T1000, v300 and relative change heatwave frequency of Figure 3 in the main manuscript but for the two other topography experiments (Figures S4 and S5). Figure S6 complements Figure 4 in the main text by displaying the composited time evolution of wavenumbers k=4,7 which are related to weaker anomalies than k=5,6 (shown in the main text). Figure S7 also complements Figure 4 in the main text by comparing the climatology of v300 with the total v300 field during amplification events of wavenumbers 5 and 6. The temporal evolution of the amplitudes of single zonal renumbers for the 8km, 25°N) simulation is shown in Figure S8. Finally, the duration of the wave amplification events is shown in Figure S9 for the different model simulations. The implications of these figures are discussed in the main manuscript.

Methods

Phase-locking index

We use the meridional wind at 300 hPa (v300) to identify Rossby waves in the midlatitude troposphere. To quantify phase-locking we define the index δ_m as the narrowest window in the phase space that contains half of the probability density. Numerically we start with a window of width $\delta = \pi$ and calculate the probability density for all the possible windows of width *delta*:

$$P(\delta, \Phi_0) = \int_{\Phi_0}^{\Phi_0+\delta} p(\Phi) d\Phi \qquad (1)$$

where $\Phi_0 \in [-\pi, \pi]$ is the left limit of the integration window and $p(\Phi)$ is the normalized probability density. Then we identify the window that contains the maximum probability $P(\delta, \Phi_0)$. If $\max[P(\delta, \Phi_0)] > 0.5$ then we decrease δ by a small amount $(2\pi/100)$ and repeat the procedure until we obtain $\delta = \delta_m$, where δ_m corresponds to the minimum value of δ, for which $\max(P(\delta_m, \Phi_0)) > 0.5$. Note that due to the size of the bins we choose, the value of δ_m has an accuracy of $\pi/50 \approx 0.063$ radians.

We also quantify the uncertainty in the δ_m value by applying a bootstrapping methodology. We select 1000 different random sub-samples consisting of 1079 blocks of 10 consecutive days each (10790 days per sub-sample) and calculate the value of δ_m for each of these sub-samples. The minimum size of the blocks is made in order to account for the auto-correlation of the time series. Using just a random subset, without requesting a minimum size of the blocks, leads to an underestimation of the uncertainty of the δ_m value. Each of the 1000 estimates of δ_m represent a probability distribution function which is used to estimate the maximum and minimum values of the distribution of δ_m. These limits are shown in Figure S3 and represent the uncertainty in δ_m in our model experiments.

Hayashi spectra

To estimate the phase speed c of the waves, the frequency - wavenumber spectra of the unfiltered v300 daily anomalies are computed for each latitudinal band using a 2-dimensional FFT. Note that for this analysis we do not use the 7-day running mean averaging as in the the previous analysis in order to represent the entire spectrum of

waves. The Hayashi spectrum (Hayashi, 1979) represents the power density spectrum of v300 in the phase speed (c) - wavenumber (k) phase space. We use the original method by Randel and Held (1991) to linearly interpolate from the wavenumber - frequency to the wavenumber - phase speed space, using a grid of resolution $\Delta c = 0.3$m/s. The obtained values are multiplied by $k/a\cos\theta$ to conserve the total power, where a is the Earth's radius and θ is latitude. Finally, we average over the the 30-60°N latitudinal band. See Domeisen, Martius, and Jiménez-Esteve (2018) and Riboldi, Rousi, D'Andrea, Rivière, and Lott (2021) for an application of this diagnostic.

References

Domeisen, D. I. V., Martius, O., & Jiménez-Esteve, B. (2018, feb). Rossby Wave Propagation into the Northern Hemisphere Stratosphere: The Role of Zonal Phase Speed. *Geophys. Res. Lett.*, *45*(4), 2064–2071. Retrieved from http://doi.wiley.com/10.1002/2017GL076886 doi: 10.1002/2017GL076886

Hayashi, Y. (1979). A Generalized Method of Resolving Transient Disturbances into Standing and Traveling Waves by Space-Time Spectral Analysis. *J. Atmos. Sci.*, *36*(6), 1017–1029. Retrieved from http://journals.ametsoc.org/doi/abs/10.1175/1520-0469(1979)036{\%}3C1017:AGMORT{\%}3E2.0.CO;2 doi: 10.1175/1520-0469(1979)036⟨1017:agmort⟩2.0.co;2

Randel, W. J., & Held, I. M. (1991). *Phase speed spectra of transient eddy fluxes and critical layer absorption* (Vol. 48) (No. 5). Retrieved from http://journals.ametsoc.org/doi/abs/10.1175/1520-0469{\%}281991{\%}29048{\%}3C0688{\%}3APSSOTE{\%}3E2.0.CO{\%}3B2 doi:

10.1175/1520-0469(1991)048⟨0688:PSSOTE⟩2.0.CO;2

Riboldi, J., Rousi, E., D'Andrea, F., Rivière, G., & Lott, F. (2021). Circumglobal rossby wave patterns during boreal winter highlighted by wavenumber/phase speed spectral analysis. *Weather and Climate Dynamics Discussions*, *2021*, 1–26. Retrieved from https://wcd.copernicus.org/preprints/wcd-2021-59/ doi: 10.5194/wcd-2021-59

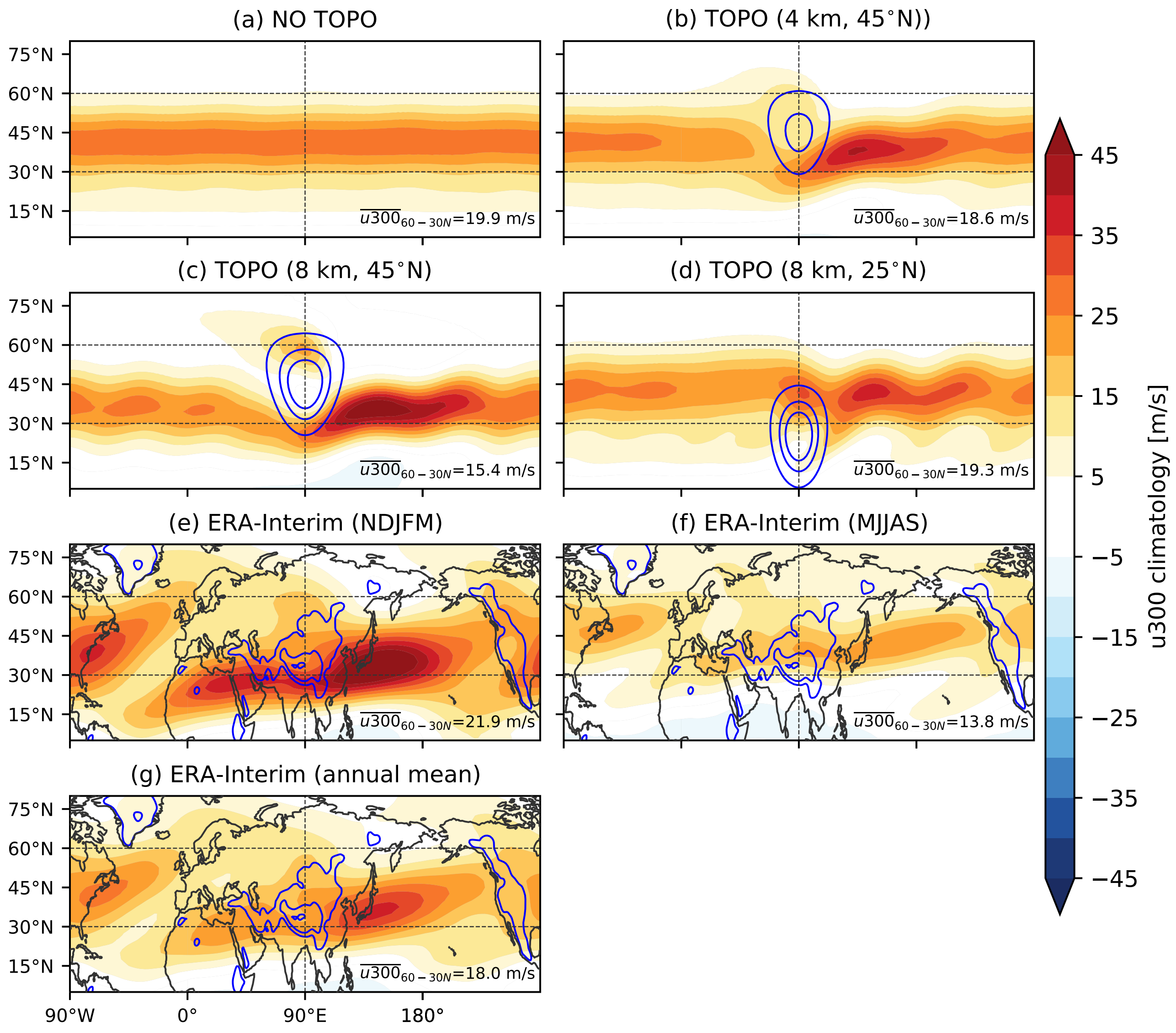

Figure S1. Climatology of zonal wind at 300 hPa (u300) (a-d) for the idealized ICON simulations (see section 2.1 in the main text), and for (e) November to March (NDJFM), (f) May to September (MJJAS) and (g) annual mean for the ERA-Interim (1979-2019) climatology. Blue contours display the topographic elevation (1, 3 and 5 km levels are shown). Topography (blue contours) has been smoothed for ERA-Interim to better capture the large-scale features. The area-weighted zonal mean of u300 between 30 to 60°N is displayed in the bottom right corner of each panel.

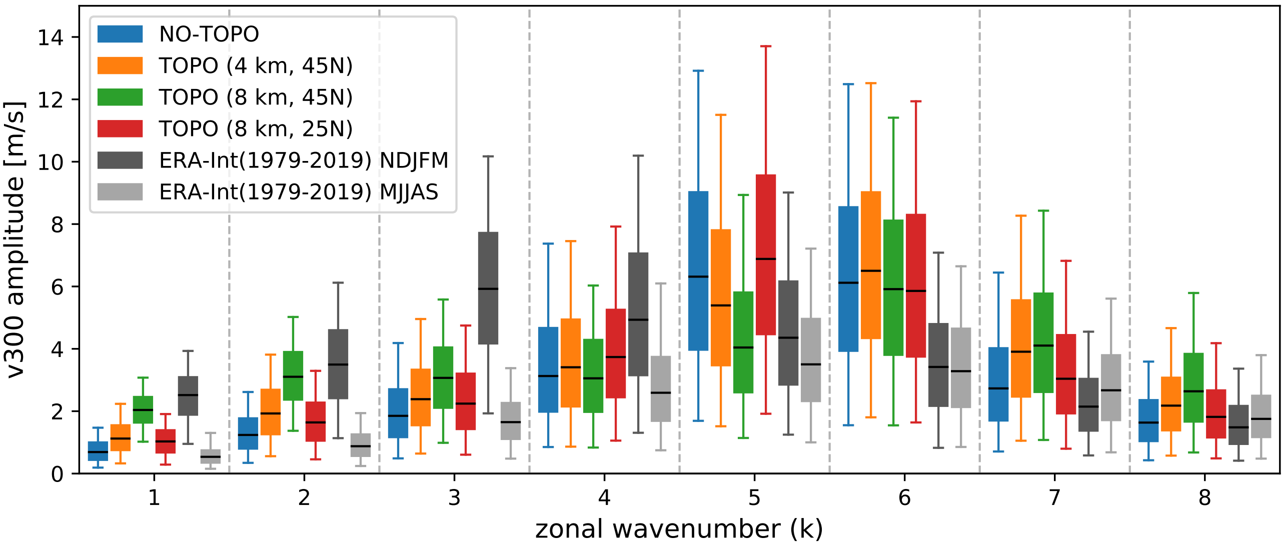

Figure S2. Box-plot of the distribution of the 7-day running mean wave amplitude of v300 for zonal wavenumbers $k = 1 - 8$ (x-axis) for the different model simulations described in the main text and for ERA-Interim reanalysis (see legend for color code). The upper and lower limits of each box represent the 75th and 25th percentiles, while whiskers represent the 95th and 5th percentiles, respectively. The median is indicated as a horizontal black line.

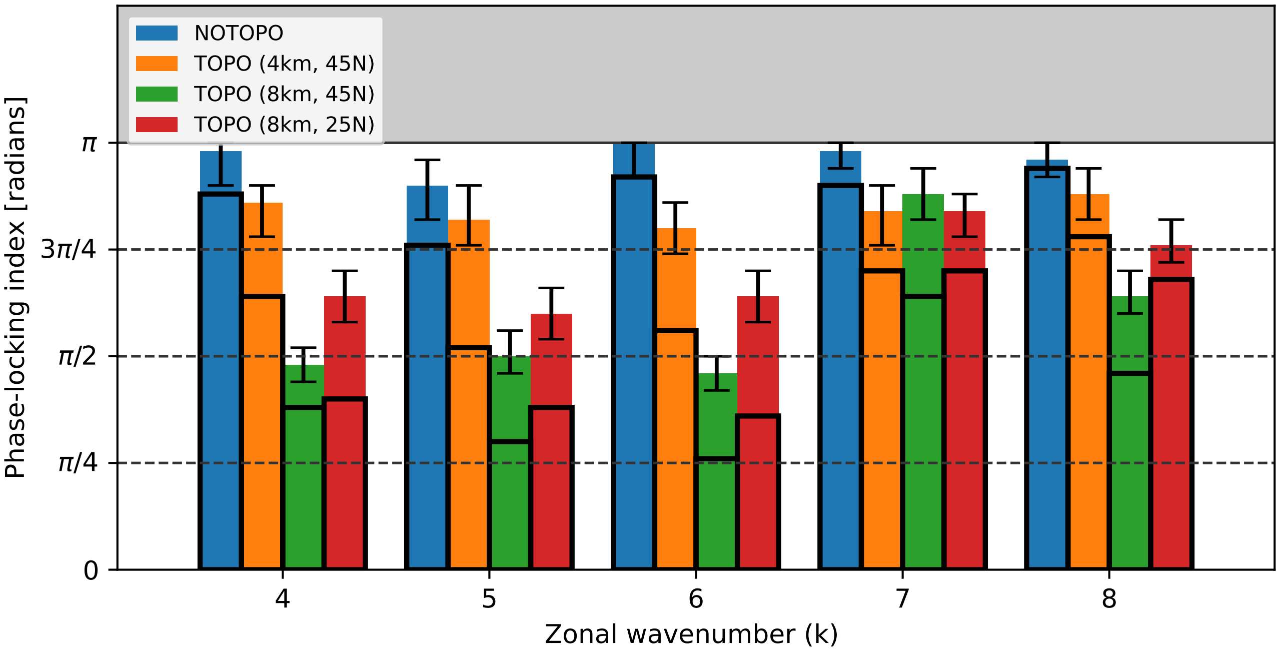

Figure S3. Phase locking index (δ_m) for zonal wavenumbers k=4-8 (x-axis) and different model simulations (shown as different colors in legend). Filled columns represent the value of the phase-locking index when using all the available data (10794 days), while the black columns represent the phase locking when only high-amplitude events (A_k >1.5SD) are considered for the calculation. Note that a value of $\delta_m = \pi$ would represent a equal probability of any phase (Φ). For the phase-locking definition please refer to the methods section in this supplementary information. The upper and lower limits of the error bars represent the maximum and minimum values, respectively, of the bootstrapped distribution using 1000 random sub-samples of 10790 days each. These consist of 1079 blocks of 10-day consecutive days respectively to account for auto-correlation. Note that due to the used binning (see methods section of this document) the uncertainty bars are not necessarily centered around the bar height.

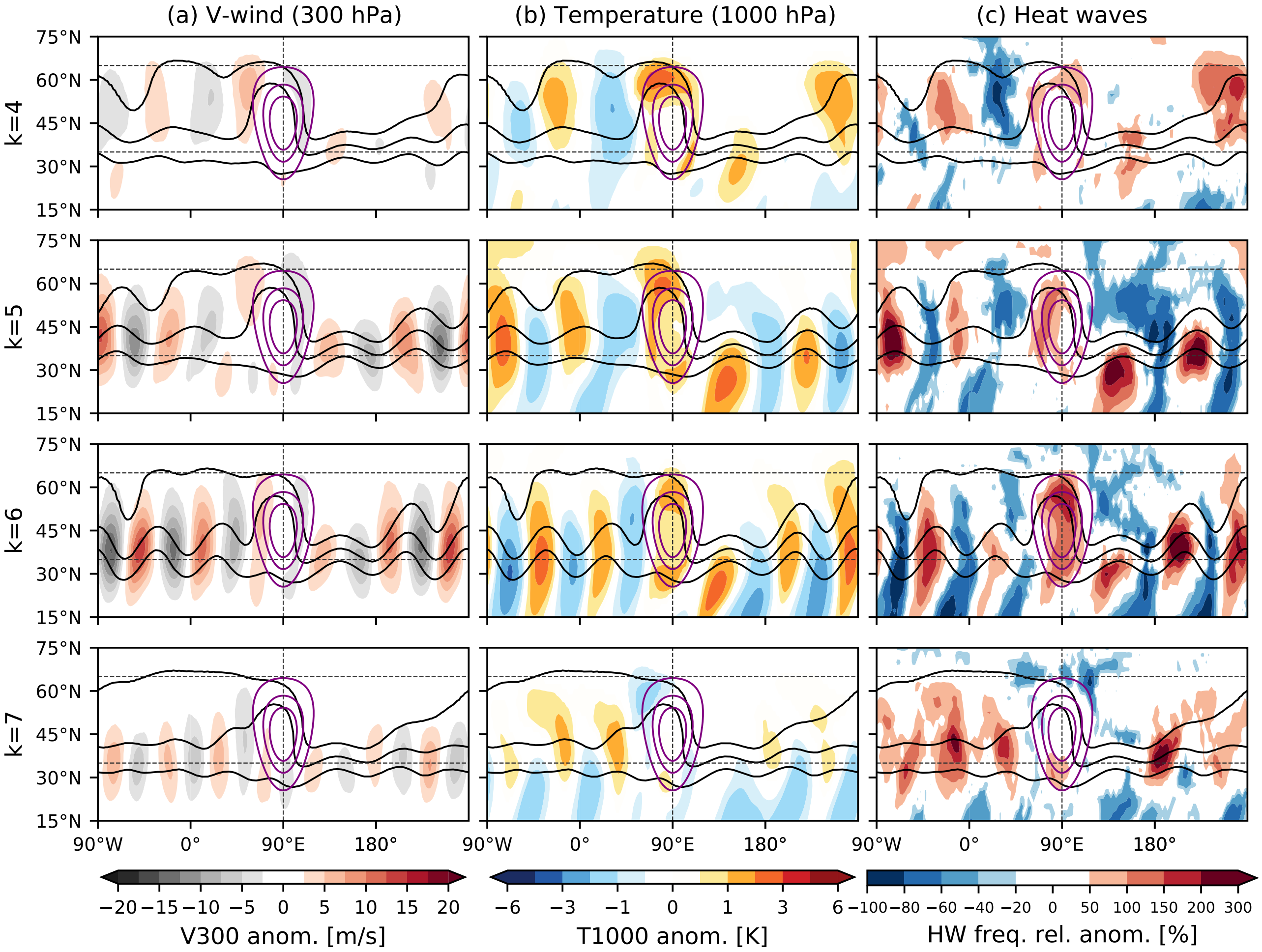

Figure S4. Same as Figure 3 in the main text but for the 8 km mountain located at 45°N.

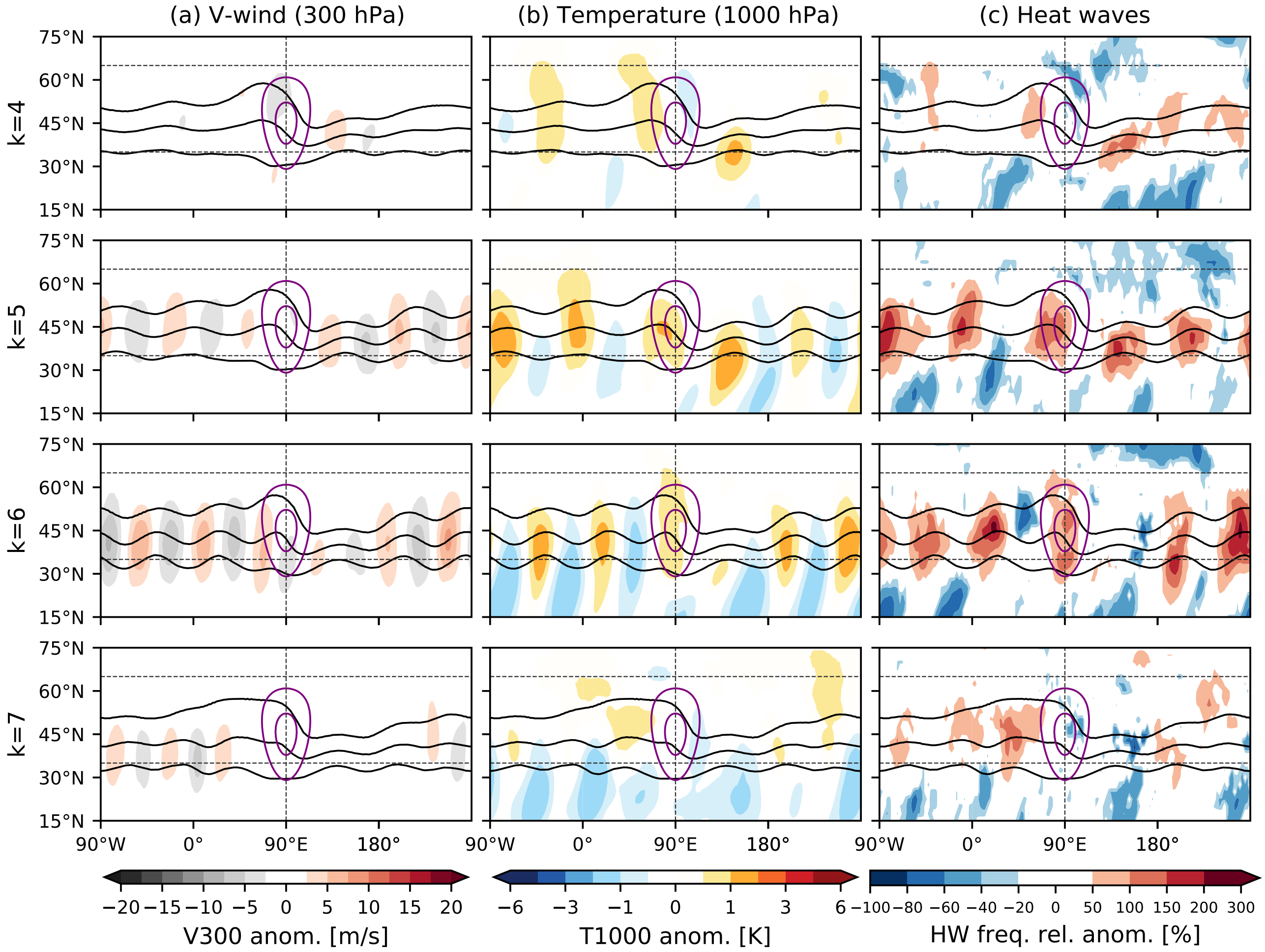

Figure S5. Same as Figure 3 in the main text but for the 4 km mountain located at 45°N.

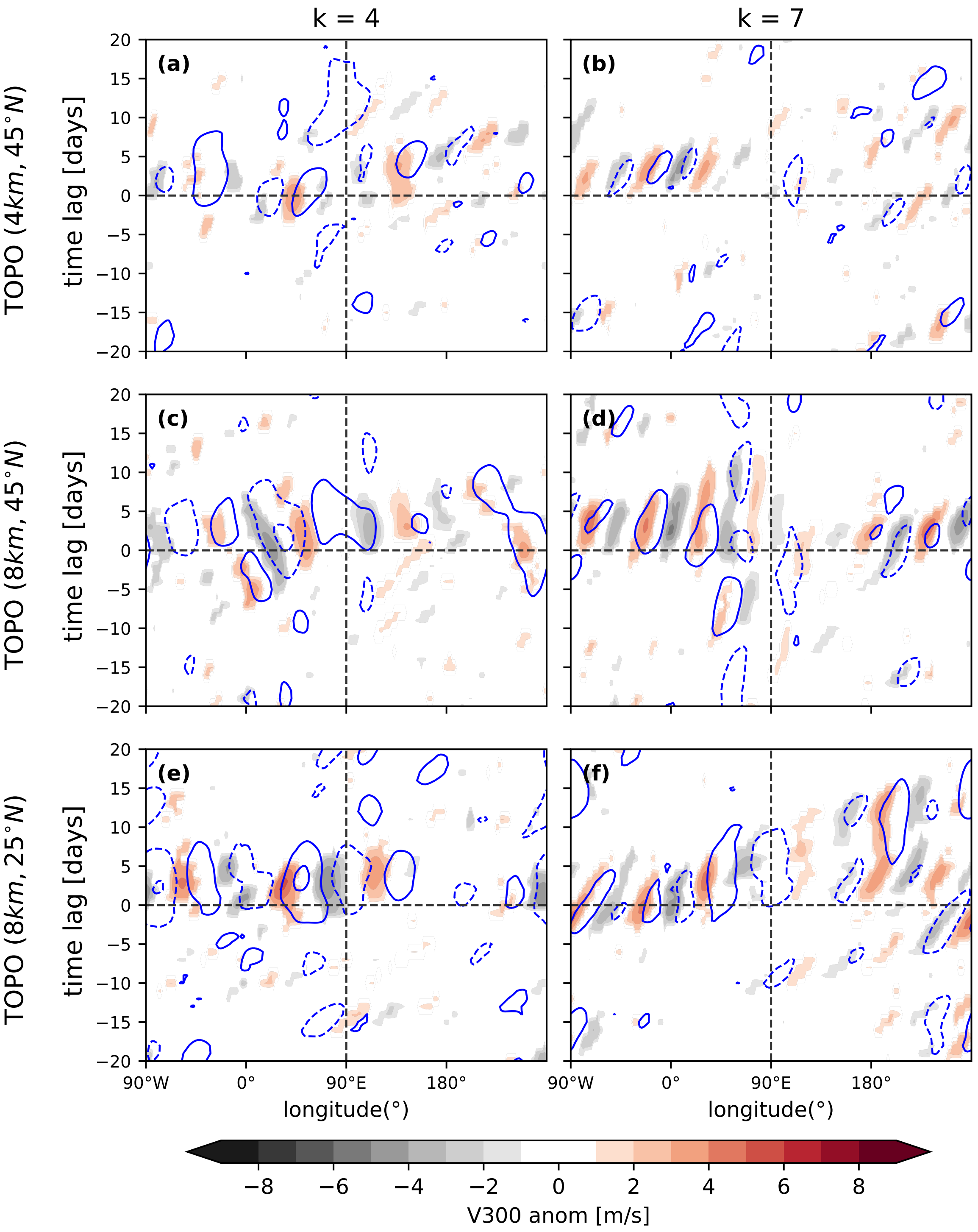

Figure S6. Same as Figure 4 in the main text but for wavenumbers k=4,7.

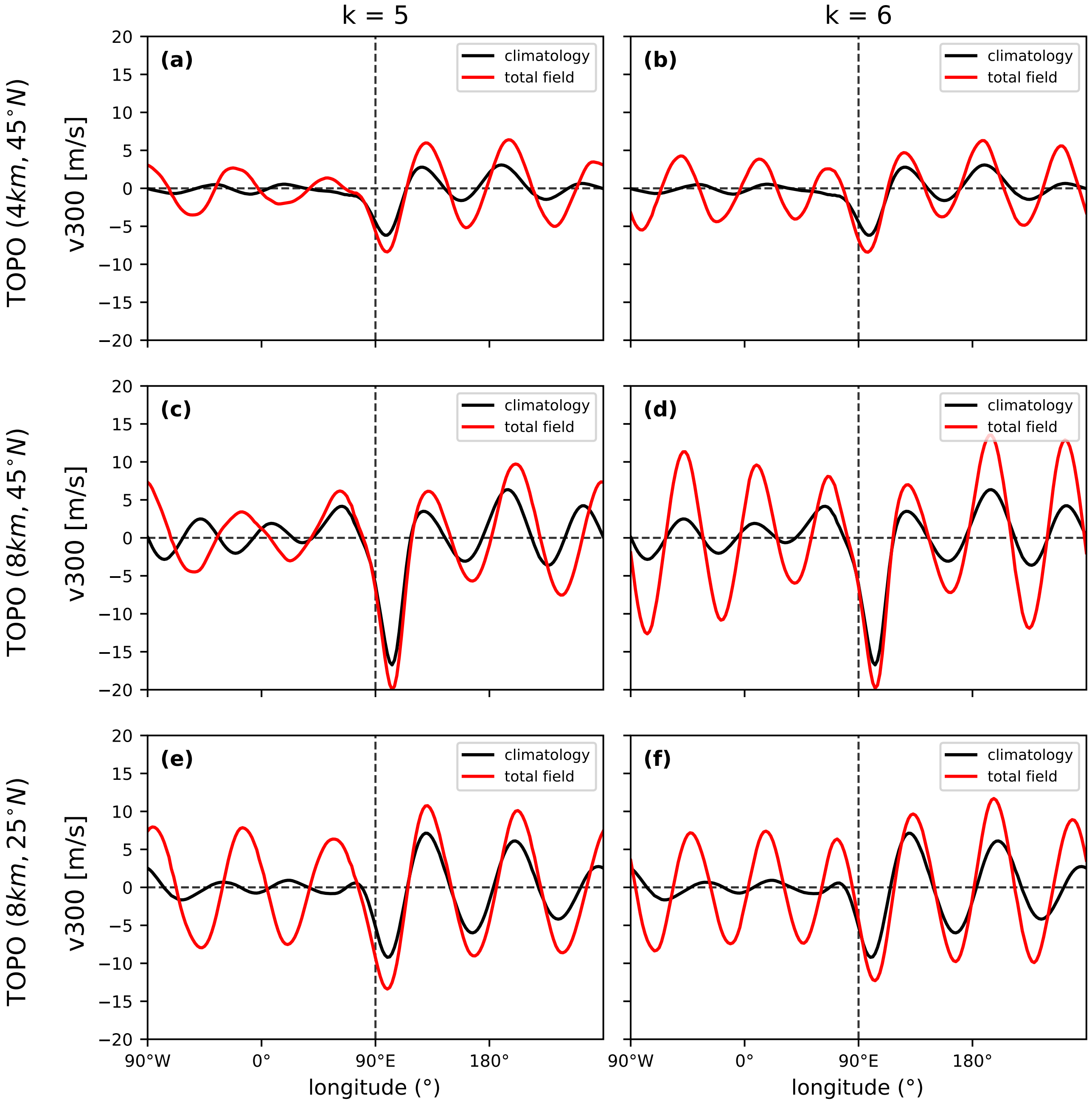

Figure S7. Climatology (black) and total field (red) of v300 averaged over 30-60°N and lags 0 to 5 days after the onset of wave amplification events (see section 2.2. in the main text) for (a,c,d) k=5 and (b,d,f) k= 6 events. (a,b) 4 km mountain at 45°N, (c,d) 8 km mountain at 45°N, and (e,f) 8 km mountain at 25°N.

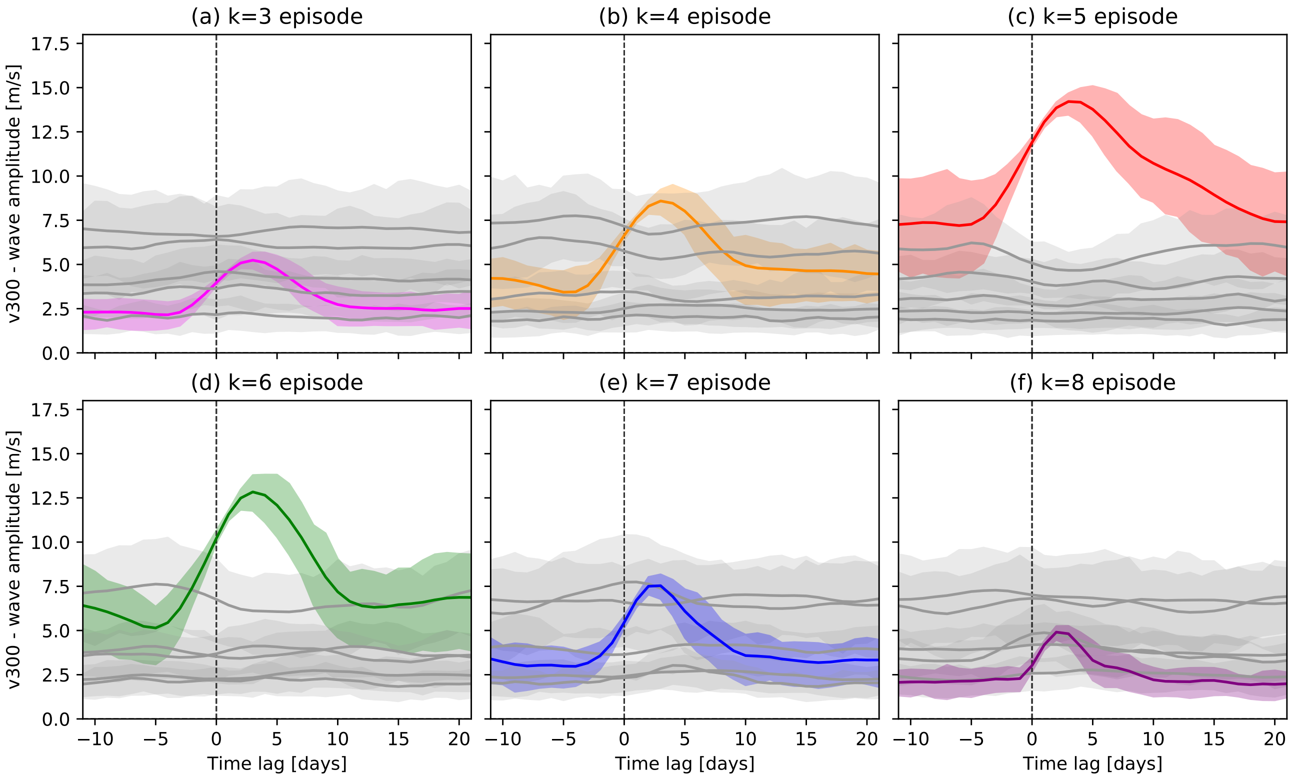

Figure S8. Temporal evolution of the v300 amplitude for different zonal wavenumbers (k) with respect to the onset of a wave amplification event for (a) k=3, (b) k=4, (c) k=5, (d) k=6 , (e) k=7, and (f) k=8 for the TOPO(8km,25°N) simulation. The average of all the events is shown as a bold line for each wavenumber. Shaded areas correspond to the 25-75th inter-percentile range. To facilitate visualization only the amplitude of the wavenumber associated with the wave amplification event is shown in color, while all the other wave amplitudes are shown in grey. Note that qualitatively similar results are obtained when reproducing this figure for the other of topographic forcings shown in this paper.

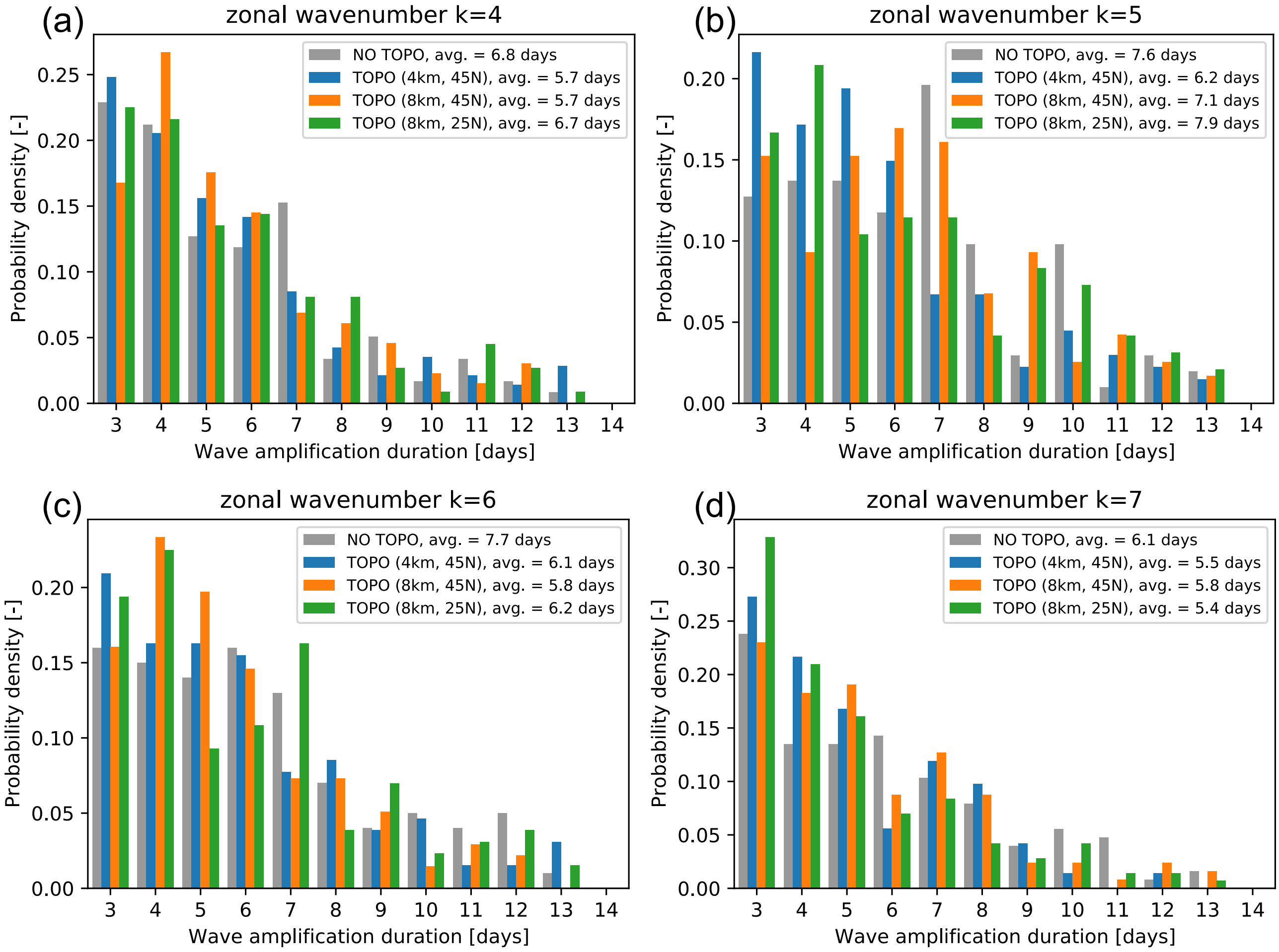

Figure S9. Histograms of duration (in days) of the wave-amplification events ($A_k > 1.5$ standard deviations for at least 3 days) for (a) k=4, (b) k=5, (c) k=6, (d) k=7. Each color represents a different model simulation (see legend). The average event duration for each zonal wavenumber and model simulation is given in the respective legend.
